# Translating neurogenomics: deconvoluting complex brain disorders

**DOI:** 10.1186/s13073-018-0517-6

**Published:** 2018-01-30

**Authors:** Rabia Begum

**Affiliations:** Genome Medicine, BMC, London, UK

Recent advances in neurogenomics have allowed exploration of the brain on a previously unprecedented scale. The development of genomic technologies combined with neurobiology tools has given rise to discoveries that have transformed our understanding of brain connectivity [1, 2] and the transition to complex brain disorders. An important example that has shaped our understanding of brain wiring is the identification of neurotoxic reactive astrocytes that are induced by activated microglia, which can result in the death of neurons and oligodendrocytes [3]. These aberrant astrocytes are abundant in amyotrophic lateral sclerosis, Alzheimer’s, Parkinson’s, and Huntington’s diseases, and thus might serve a central role in these distinct neurodegenerative diseases [3]. Therefore, tissue-level genomics must be fine-tuned to single-cell resolution in conjunction with molecular neurobiology to capture genetic complexity, the molecular basis of normal function, and the transition to disease [4].

In this special issue on Disease Neurogenomics, we sought to capture novel insights into the impact of genetic and genomic variation with a special focus on neurodevelopmental, neuropsychiatric, and neurodegenerative diseases. The articles published in this issue present advances that bridge the gap between genomics and neurobiology to improve our understanding of disease risk and the translational potential for diagnostic and therapeutic opportunities. In the study by Kauwe and colleagues, which formed part of the Alzheimer’s Disease Neuroimaging Initiative, the authors identified rare variants in *RAB10* that provide resilience to Alzheimer’s disease in ‘high-risk’ individuals, suggesting that this gene could be a promising therapeutic target. The approach used in this study could be repurposed to identify rare variants associated with resilience to other complex diseases [5].

Neurodevelopmental and neuropsychiatric disorders represent a spectrum of heterogeneous disorders with degrees of shared genetic etiology [6, 7]. Hakonarson and colleagues [6] conducted the first large-scale meta-analysis to identify shared copy number variants (CNVs) across schizophrenia, bipolar disease, autism spectrum disorders, attention deficit hyperactivity disorder, and depression. Novel associations of *DOCK8*/*KANK1* duplications were shared by the five disorders, providing a CNV map that could be used to elucidate common molecular pathways across these heterogeneous phenotypes [6, 7].

Although rare CNVs are known to play a role in schizophrenia, their impact in the context of intelligence quotient (IQ) within these patients is unclear. Bassett and colleagues showed that the burden of pathogenic CNVs in schizophrenia differs between IQ subgroups in a community sample of adults [8]. They report that the highest number of pathogenic CNVs was identified in individuals with schizophrenia in whom IQ is most compromised. The results could have considerable implications for clinical practice, suggesting that patients with a dual diagnosis of schizophrenia and intellectual disability should be prioritized for testing.

The functional impact of de novo mutations are of particular interest in neurodevelopmental disorders given their prevalence in patients. Eichler and colleagues review the state of neurodevelopmental disorders in the context of de novo mutations (DNMs), including CNVs, highlighting patterns of DNM enrichment and cell type-specific convergence in the brain [9]. The authors also provide guidance on DNM interpretation and their potential for informing diagnostics.

In order to further understand the overlap between schizophrenia and four other neurodevelopmental disorders, Stahl and colleagues developed an extended pipeline called extTADA to analyze rare exonic variants from whole exome sequence data. This pipeline could derive insights into the genetic complexity of rare variation in schizophrenia versus other neurodevelopmental disorders and novel risk genes [10].

Mapping the genetic architecture of complex diseases is being intensively investigated via multiple routes. Neuroimaging genomics is one of these routes, which aims to map genetic variation to brain structural phenotypes. Jahanshad and colleagues highlight emerging pathways associated with neural phenotypes and discuss the need for refined brain mapping to localize variation to specific tissue layers and subfields [11].

Risk prediction in these diseases is difficult because of the polygenic nature of complex disorders; however, application of polygenic risk scores (PRS) is a widely used approach that has predictive power for case-control status [12]. Gelernter and colleagues used a high-resolution polygenic score approach and Mendelian randomization to identify a putative causal relationship between genetically determined female body shape and posttraumatic stress disorder [13]. PRS have thus far shown promise as a potential tool for risk prediction. The translational potential of PRS in polygenic medicine is discussed in a Comment by Lewis and Vasso [12].

Together, these collective efforts further our understanding of disease risk and the underlying neurobiology of complex brain disorders. We must continue in the direction of integrated, interdisciplinary, and open research in order to uncover the mechanisms by which genetic and genomic variation influence disease initiation and progression in these highly heterogeneous conditions; such a direction will only improve our ability to stratify patients to develop more informed diagnostic and therapeutic approaches.

## Abbreviations

CNV: Copy number variant
DNM: De novo mutation
IQ: Intelligence quotient
PRS: Polygenic risk scores
